# Post-Exercise Whole Body Cryotherapy (−140 °C) Increases Energy Intake in Athletes

**DOI:** 10.3390/nu10070893

**Published:** 2018-07-12

**Authors:** Chihiro Kojima, Nobukazu Kasai, Chika Kondo, Kumiko Ebi, Kazushige Goto

**Affiliations:** Graduate School of Sport and Health Science, Ritsumeikan University, Kusatsu, Shiga 5258577, Japan; sh0007ek@ed.ritsumei.ac.jp (C.K.); sh0004ef@ed.ritsumei.ac.jp (N.K.); chii.2727@gmail.com (C.K.); ab@fc.ritsumei.ac.jp (K.E.)

**Keywords:** appetite regulation, exercise–induced anorexia, appetite-regulating hormones, cold environment, energy balance

## Abstract

PURPOSE: The purpose of the present study was to investigate the effect of whole-body cryotherapy (WBC) treatment after exercise on appetite regulation and energy intake. METHODS: Twelve male athletes participated in two trials on different days. In both trials, participants performed high-intensity intermittent exercise. After 10 min following the completion of the exercise, they were exposed to a 3-min WBC treatment (−140 °C, WBC trial) or underwent a rest period (CON trial). Blood samples were collected to assess plasma acylated ghrelin, serum leptin, and other metabolic hormone concentrations. Respiratory gas parameters, skin temperature, and ratings of subjective variables were also measured after exercise. At 30 min post-exercise, energy and macronutrient intake were evaluated during an ad libitum buffet meal test. RESULTS: Although appetite-regulating hormones (acylated ghrelin and leptin) significantly changed with exercise (*p* = 0.047 for acylated ghrelin and *p* < 0.001 for leptin), no significant differences were observed between the trials. Energy intake during the buffet meal test was significantly higher in the WBC trial (1371 ± 481 kcal) than the CON trial (1106 ± 452 kcal, *p* = 0.007). CONCLUSION: Cold exposure using WBC following strenuous exercise increased energy intake in male athletes.

## 1. Introduction

An acute bout of exercise has been shown to cause a temporary suppression of appetite and energy intake [1,2,3] although some inconsistent results exist [4]. This suppression phenomenon is regarded as “exercise-induced anorexia” [5], which is associated with transient changes in appetite-regulating hormones (e.g., ghrelin (an appetite stimulating hormone [6]), peptide YY (PYY, an appetite-suppressing hormone [7]), and glucagon-like peptide-1 (GLP-1, an appetite-suppressing hormone, [8])). Exercise-induced reduction of energy intake is beneficial for health promotion and prevention of obesity in general, but it can have a negative effect on athletes. The impact of exercise on appetite regulation in competitive athletes has not been fully explored. Previous studies evaluating well-trained athletes found that a single bout of exercise significantly reduced ghrelin concentrations, with lower subjective ratings of hunger, elevated PYY_3-36_ and GLP-1 concentrations, and subsequently reduced energy intake during post-exercise meals [9,10,11]. Howe et al. [11] indicated that appetite suppression occurs during a specific time window (e.g., 30–40 min post-exercise) when the body is metabolically primed for anabolic processes if fuel and nutrients are received. Therefore, it is essential for athletes to reserve optimal energy intake after exercise. Additionally, faster recovery from exercise-induced appetite suppression may facilitate exercise recovery and improve overall performance. 

Loss of appetite and reduced energy intake following exercise may be influenced by environmental factors, such as temperature and hypoxic conditions. In particular, exercise in cold environments promotes hunger and feeding behavior [12,13,14,15]. Wasse et al. [16] observed an increase in energy intake following running in a cold environment. Crabtree and Blannin [14] found that a treadmill walk in a cold environment promoted post-exercise energy intake. Additionally, cold water immersion during exercise or post-exercise recovery also increased energy intake [12,17]. Augmented post-exercise energy intake in cold environments may be explained by elevation of ghrelin and reduction of leptin. Tomasik et al. [18] found that 30 min of cold exposure significantly increased ghrelin levels. Post-exercise ghrelin elevation was also greater after exercise in a cold environment as compared to a neutral environment [14]^.^ Furthermore, Zeyl et al. [19] found that cold water immersion (18 °C) lowered leptin concentrations. Taken together, exposure to cold appears to promote appetite and energy intake during the post-exercise period. 

Recently, whole -body cryotherapy (WBC) has received increased attention as an aid to post-exercise recovery [20,21]. WBC consists of a 2–4 min exposure to extreme cold air (−100 to −195 °C) in a cabin [21,22,23,24]. Several studies have shown that WBC attenuated exercise-induced muscle damage and inflammatory responses [20,23,25] and promoted recovery of exercise performance [22,24]. Taking into consideration an orexigenic effect of the cooling body, the use of WBC is likely to stimulate appetite during the post-exercise period. To date, no study has evaluated the impact of post-exercise WBC on energy intake during the subsequent meal. The purpose of the present study was to investigate the effect of WBC on post-exercise appetite regulation. We hypothesized that plasma ghrelin concentration would be increased and serum leptin concentrations would be decreased, leading to enhanced post-exercise energy intake after WBC treatment following strenuous exercise. 

## 2. Materials and Methods

### 2.1. Participants

Twelve male college athletes (mean ± standard deviation (SD) age: 20.5 ± 1.1 years, height: 174.2 ± 4.9 cm, weight: 65.6 ± 6.4 kg, BMI (body mass index): 21.5 ± 1.4 kg/m^2^) were recruited from the same university track and field club. The athletes trained on the track approximately five days per week (2.5 h/day). The study was conducted during the off-season (at the beginning of the pre-season). As feeding behavior is thought to be influenced by psychological factors, the participants were not completely informed of the purpose of this study. They were told that the purpose was to determine the effect of WBC on post-exercise recovery and taste perception. Participants were informed of the experimental procedures and risks of the study, and provided written informed consent. This study was approved by the Ethics Committee for Human Experiments at the Ritsumeikan University, Japan.

### 2.2. Experimental Overview

Participants completed two trials on different days using a cross-over design: exposure to WBC (WBC trial) or rest (CON trial) following strenuous exercise. Each trial was separated by a minimum of two weeks, and the order of trials was counter-balanced (six subjects conducted WBC trial initially, and remaining six subjects started with the CON trial). Metabolic and hormonal responses, ad libitum energy and macronutrient intake, and ratings of subjective variables were compared between the two trials. 

### 2.3. Experimental Protocol in WBC Trial and CON Trial

On the day prior to the trials, participants were instructed to replicate the amount of training and meals. For each trial, participants visited the laboratory following an overnight fast. Baseline measurements of body composition and subjective feelings (using visual analogue scales (VAS)) were conducted. After 20 min of rest, skin temperature was measured and a baseline blood sample was collected. Participants then began a repeated sprint exercise using an electromagnetically braked cycle ergometer (Power Max VIII; Konami Corporation, Tokyo, Japan). The exercise consisted of four components: (1) Warm-up (5 min of pedaling at 80–100 rounds per minute (rpm) and 3 × 3-s periods of maximal pedaling); (2) ten seconds of maximal pedaling at 1.5% of body weight; (3) three successive sets of 5 × 6-s maximal pedaling with 24 s of rest at 7.5% of body weight (8-min rest period between sets); and (4) two periods of maximal pedaling for 20 s, with 5 min rest, at 7.5% and 5.0% of body weight, respectively. A 5–10 min rest period was inserted between components. The above protocol was designed to improve anaerobic power output in 100–200 m track and field sprinters; we previously confirmed that the training increases 10-s maximal power output and repeated sprint ability [26]. Environmental conditions during the exercise session were controlled at 22 °C and 40% humidity. 

After completing exercise, blood samples were collected followed by measurements of subjective feelings. Skin temperature was monitored during the post-exercise period. At 10 min after exercise, participants underwent 3 min of WBC (−140 °C) treatment (WBC trial) or rest at room temperature (CON trial). The WBC treatment was conducted using a specially designed cryo-cabin (Cryo Shower, Saraya Co., Ltd., Osaka, Japan). The cabin was filled with cold (dry) air made by gaseous nitrogen. During the 3 min of WBC treatment, temperature within the cabin and oxygen concentration in the room were monitored continuously. Duration and temperature of the WBC treatment were determined based on previous studies [24,27,28]. To avoid cold injury during the WBC treatment, participants were instructed to walk slowly around the chamber and to flex and extend elbows and fingers. They were also permitted to wear shoes and socks during the treatment. 

At 30 min after exercise (which corresponds to 17 min after completing WBC), resting respiratory gas samples were collected for 5 min. Heart rate was monitored every 5 s using a Polar RCX5 heart rate monitor (Polar Electro Oy, Kempele, Finland). Blood samples were subsequently obtained. After the series of measurements, ad libitum energy and macronutrient intake were evaluated during the buffet meal test. 

### 2.4. Measurements

#### 2.4.1. Blood Parameters

On trial days, participants arrived at the laboratory at 8:00 or 9:00 a.m. following an overnight fast. After resting for 20 min, baseline blood samples were obtained. Additional blood samples were collected immediately after exercise (prior to WBC) and at 30 min after exercise (17 min after completing WBC). A series of blood samples were collected using venipuncture. Serum and plasma samples were centrifuged for 10 min at 4 °C and stored at −80 °C until analysis. Blood glucose, lactate, plasma acylated ghrelin, serum leptin, and insulin concentrations were determined. Blood glucose and lactate concentrations were measured immediately after blood collection using a glucose analyzer (Free style, Nipro Co., Osaka, Japan) and a lactate analyzer (Lactate Pro, ARKRAY Co., Kyoto, Japan), respectively. For plasma ghrelin, blood was drawn into a chilled tube containing EDTA, dipeptidyl peptidase-4 (DPP-V) inhibitor, and a protease and esterase inhibitor to avoid inactivation of ghrelin. After obtaining plasma samples by centrifugation at 4 °C, hydrochloric acid (1 mmol/L) was added in a microtube for analysis of ghrelin, as suggested by the manufacturer. Plasma ghrelin concentration was measured using an enzyme-linked immunosorbent assay (ELISA) kit (Mitsubishi chemical Medicine Co., Tokyo, Japan). The intra-assay coefficient of variation (CV) was 5.7%. Serum leptin concentration was also measured using an ELISA kit (R&D Systems, Inc., Minneapolis, MN, USA). The intra-assay CV was 3.8%. Serum insulin concentration was measured using chemiluminescent immunoassay at a clinical laboratory (SRL Inc., Tokyo, Japan). The intra-assay CV was 3.1%.

#### 2.4.2. Subjective Feelings of Appetite, Fatigue, and Muscle Soreness

Ratings of hunger, appetite, prospective food consumption, fullness, fatigue, and muscle soreness were evaluated using a 100-mm VAS [29,30] before exercise, immediately after exercise, 15 min after exercise (at 2 min after completing WBC), 30 min after exercise (at 17 min after completing WBC), and after the ad libitum buffet meal.

#### 2.4.3. Respiratory Parameters

At 22 min post-exercise (9 min after completing WBC), respiratory gas samples were collected for 5 min from each participant while seated. Oxygen uptake ($\dot{V}$O_2_), carbon dioxide output ($\dot{V}$CO_2_), and minute ventilation ($\dot{V}$E) were determined with an automatic gas analyzer (AE310S, Minato Medical Science Co., Ltd, Tokyo, Japan). Values were averaged every 30 s. During the 5 min sampling period, data from the final 2 min were utilized for analysis.

#### 2.4.4. Skin Temperature

Skin temperature was monitored every 5 s using an NT logger monitor N543 (NIKKISO-Therm Co., Ltd, Tokyo, Japan) before and after exercise. A probe was placed on the thigh at the midpoint between the great trochanter and knee joint (the rectus femoris muscle). The measurements before exercise lasted for 3 min. Skin temperature was monitored continuously from 3 min after exercise completion to the end of the buffet meal.

#### 2.4.5. Ad Libitum Buffet Meal

The ad libitum buffet meal test began 30 min after exercise (17 min after completing WBC) to evaluate energy and macronutrient intake. The participants were not informed of the elapsed time during the test, but the test finished within 30 min. All participants were instructed to “eat until they felt comfortable satiety”. To avoid any psychological influence on food selection, they ate in isolation without talking. The ad libitum meal consisted of 18 items: bread, raisin bread, jam, ham, boiled chicken, sausage, boiled egg, potato salad, corn soup, milk, yogurt, cheese, orange, banana, green tea, orange juice, and vegetable juice. The food items, place and time of the day for buffet meal test were identical between two trials. Energy intake was calculated by measuring the weight of energy intake consumed. In short, it was determined by counting the number of plates (the number of calories per plate was predetermined) and weighing the remaining food after eating. A dietary analysis program (Excel Eiyou-kun ver. 6.0, Kenpakusha, Tokyo, Japan) was used to calculate energy and macronutrient intakes. The procedures were established in previous studies [9,10].

### 2.5. Statistical Analysis

Data are expressed as means ± SD. The time course of changes in skin temperature, blood parameters and subjective feelings were compared using a two-way repeated-measures analysis of variance (ANOVA) to determine interaction (trial × time) and main effects (trial, time). If ANOVA revealed a significant interaction or main effect, the Tukey–Kramer test was used. Energy intake and respiratory gas parameters were compared between the two conditions using a paired *t*-test. A *p* value < 0.05 was used to determine statistical significance. 

## 3. Results

### 3.1. Exercise Performance

Power output during exercise was successfully recorded for 11 of 12 participants (data from one participant was missing due to exercise equipment failure). Total power output (calculated by summing power output during all sets of exercises) did not significantly differ between the WBC (11,405 ± 1557 W) and CON trials (11,298 ± 1540 W, *p* = 0.257).

### 3.2. Skin Temperature

**Figure 1:**
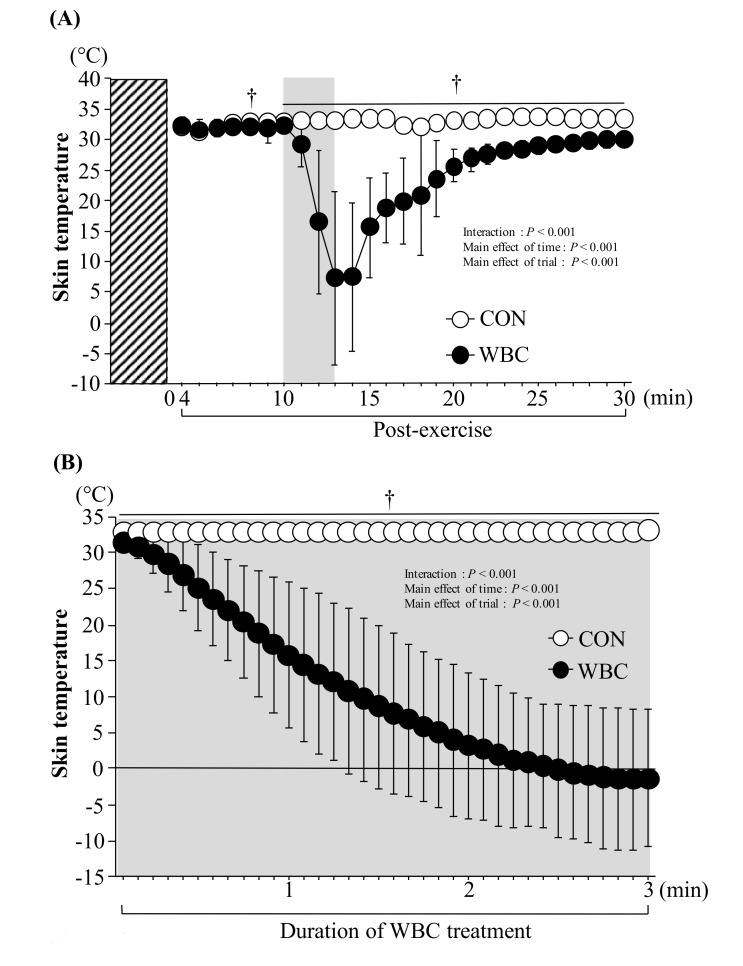
Skin temperature during post-exercise period (**A**) and during the WBC period (**B**). Values are means ± standard deviation (SD) (*n* = 12). †: *p* < 0.05 vs. CON. Gray bar shows duration (3 min) of WBC treatment. A diagonal box indicates duration of exercise. WBC: whole-body cryotherapy; CON: rest period.

Before exercise, skin temperature at rest did not differ between the WBC (31.6 ± 0.8 °C) and CON trials (31.4 ± 0.9 °C, *p* > 0.05). After WBC treatment, skin temperature was significantly lower in the WBC trial compared with the CON trial, which remained lower until the start of the buffet meal test (*p* < 0.05, Figure 1A). At the start of the meal, participants had significantly lower skin temperature in the WBC trial (29.9 ± 1.0 °C) than in the CON trial (33.2 ± 0.9 °C, *p* < 0.001, Figure 1A). Figure 1B shows the time course of changes in skin temperature during the 3-min WBC treatment. The WBC treatment caused marked reduction in skin temperature (*p* < 0.001 vs. CON), and a significant interaction (time × trial) and main effects of time and trial were observed (*p* < 0.001).

### 3.3. Ratings of Appetite, Fatigue and Muscle Soreness

Table 1 shows the time-course change in ratings of appetite, fatigue, and muscle soreness. Prior to exercise, no significant differences between trials were observed. Ratings of fatigue revealed a significant main effect of time (*p* < 0.001), with no significant difference between the trials. For muscle soreness, there was a significant main effect of time (*p* < 0.001) and trial (*p* = 0.032). Additionally, the occurrence of muscle soreness was significantly higher in the CON trial than in the WBC trial at 15 and 30 min following exercise (*p* < 0.05). Ratings of hunger and prospective food consumption showed a significant main effect of time (*p* < 0.001 for both variables). However, no significant differences were observed between the two trials (*p* > 0.05). For fullness, there was a significant interaction (time × trial, *p* = 0.005) and main effect of time (*p* < 0.001). Following the meal, the rating of fullness was significantly higher in the WBC trial than in the CON trial (*p* < 0.05).

### 3.4. Respiratory Parameters and Heart Rate

Table 2 shows the changes in $\dot{V}$O_2_, $\dot{V}$CO_2_, $\dot{V}$E, and Heart rate (HR) during the post-exercise period (collected at 22–27 min after exercise completion, or 9–14 min after WBC). $\dot{V}$CO_2_, $\dot{V}$E, and HR values were significantly lower in the WBC trial than in the CON trial (*p* = 0.007 for $\dot{V}$CO_2_, *p* = 0.016 for $\dot{V}$E, *p* = 0.042 for HR). $\dot{V}$O_2_ did not differ significantly between trials (*p* = 0.196).

### 3.5. Blood Parameters

Table 3 shows the time-course change in blood glucose, lactate, and serum insulin concentrations. Before exercise, there were no significant differences between trials. All variables revealed a significant main effect of time (*p* = 0.004 for glucose, *p* < 0.001 for lactate and insulin). However, no significant difference between trials was observed for any variable (*p* > 0.05).

**Figure 2:**
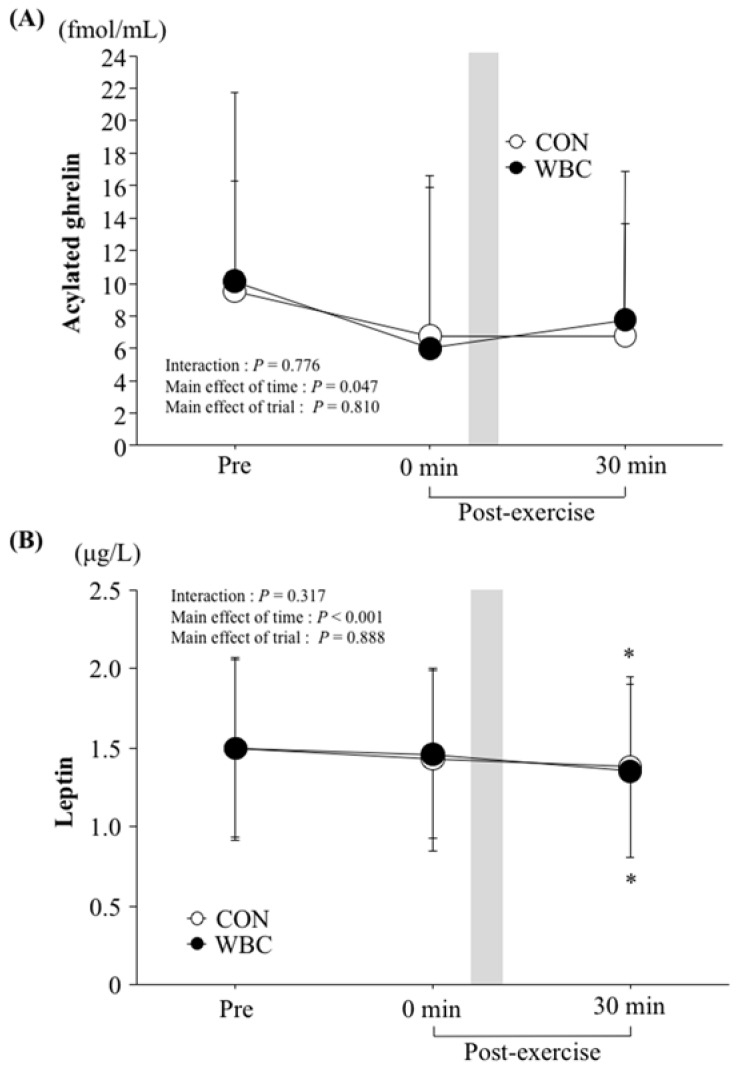
Plasma acylated ghrelin (**A**) and serum leptin (**B**) concentrations. Values are means ± SD (*n* = 12). *: *p* < 0.05 vs. pre. Gray bar shows duration (3 min) of WBC treatment. WBC: whole body cryotherapy; CON: rest period.

Figure 2 shows the time-course change in plasma acylated ghrelin (A) and serum leptin (B) concentrations. There were no significant differences in pre-exercise values between the trials. Although all variables revealed a significant main effect of time (*p* = 0.047 for acylated ghrelin, *p* < 0.001 for leptin), there was no significant difference between the two trials in any parameters.

### 3.6. Energy and Macronutrient Intakes

Duration of consumed energy intake during the buffet meal test was significantly longer in the WBC trial (26.1 ± 5.6 min) than the CON trial (20.9 ± 6.2 min, *p* = 0.011).

**Figure 3:**
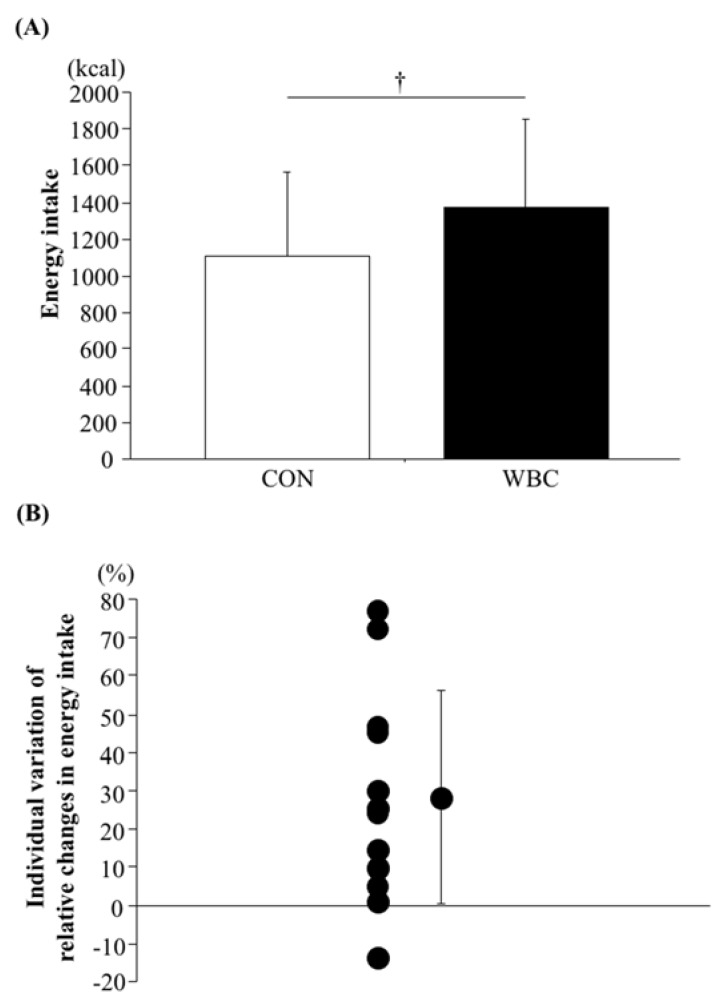
Energy intake (**A**) and individual variation of relative changes in energy intake (**B**) during ad libitum buffet meal test. The value (**B**) was calculated as relative increase (percentage of change) of energy intake in WBC trial (vs. CON trial). Above 0% (11 of 12 participants) means that energy intake was greater in the WBC trial than that in the CON trial (CON trial < WBC trial). Below 0% (1 of 12 participants) means that energy intake was greater in CON trial than that in WBC trial (CON trial > WBC trial). Values are means ± SD (*n* = 12). †: *p* < 0.05 vs. CON. WBC: whole body cryotherapy; CON: rest period.

Figure 3 shows energy intake as well as the individual variation of relative changes in energy intake during the ad libitum buffet meal test. Energy intake was significantly higher in the WBC trial (1371 ± 481 kcal) than the CON trial (1106 ± 452 kcal, *p* = 0.007). For 11 of the 12 participants, energy intake was higher in the WBC trial than the CON trial.

The weight of the consumed food did not differ significantly between trials (*p* = 0.245). The macronutrient intake ratio did not significantly differ between the WBC trial (19.8 ± 1.7% for protein, 41.5 ± 6.6% for fat, and 38.7 ± 6.6 for carbohydrate) and the CON trial (19.0 ± 3.2% for protein, 38.3 ± 7.3% for fat, and 42.7 ± 9.5% for carbohydrate, *p* = 0.245 for protein, *p* = 0.118 for fat, and *p* = 0.124 for carbohydrate).

## 4. Discussion

The purpose of this study was to determine the effect of post-exercise WBC (−140 °C for 3 min) on appetite regulation and energy intake. The novel finding was that 3 min of WBC treatment following intensive exercise significantly increased energy intake during the buffet meal test compared with the non-treatment condition. Appetite-regulating hormones (i.e., plasma ghrelin and serum leptin) were probably not involved in this effect, as there were no significant differences in these hormones before the onset of the buffet meal test. These results suggest that post-exercise WBC treatment is suitable for competitive athletes to increase post-exercise energy intake, and may facilitate recovery and improve performance.

### 4.1. Appetite-Regulating Hormones Following WBC

In the present study, ad libitum energy intake increased by 24% after WBC treatment. Additionally, 11 of 12 participants exhibited higher energy intake following WBC treatment, which suggests that inter-individual variation in the orexigenic impact of post-exercise WBC was substantially minor. We hypothesized that increased energy intake after WBC would be explained by transient changes in appetite-regulating hormones, as these hormones (e.g., ghrelin, leptin) were previously associated with augmented appetite and energy intake in cold environments [17,18,19,31]. Tomasik et al. [18] demonstrated that cold exposure (2 °C) caused higher total ghrelin concentrations compared with neutral (20 °C) and hot environments (30 °C). Cold water immersion (18 °C) has also been found to lower leptin concentrations [19]^.^ In a study by Halse et al. [17], energy intake was augmented by post-exercise water immersion (15 °C and 33 °C), which may have been influenced by lowered leptin concentration, although influence of different water temperature was not observed. Possible mechanisms for altered appetite-regulating hormones in cold climate conditions include reheating of skin temperature after water immersion [12] and increased splanchnic blood volume induced by reductions of skin temperature and skin blood flow [14]. In the present study, skin temperature was continuously monitored throughout the post-exercise period, and dropped to 7.1 ± 14.2 °C immediately after the WBC treatment. Subsequently, the reduced skin temperature was rapidly elevated to 29.9 ± 1.0 °C at 30 min following exercise (immediately before beginning the buffet meal test). Contrary to previous findings and our hypothesis, we did not find significant alterations in plasma ghrelin or serum leptin concentrations between trials at any points. Thus, increased energy intake following WBC treatment was independent of changes in appetite-regulating hormones.

### 4.2. Other Potential Mechanisms to Promote Energy Intake Following WBC

Alternatively, changes in the sympathetic and parasympathetic nervous systems may be responsible for the increase in energy intake after WBC treatment. Assessments of parasympathetic activity (measured by heart rate variability) following WBC are somewhat limited [32,33]. Although we did not determine heart rate variability, HR at 10 min after WBC was significantly lower in the WBC trial (95 ± 11 bpm) than in the CON trial (101 ± 8 bpm, *p* < 0.05). This finding is in accordance with previous studies [32,33]. Cold stimulation, including WBC treatment, triggers peripheral vasoconstriction, leading to a blood volume shift from the superficial part (skin) toward the core [34]. The increased blood volume in deep blood vessels promotes venous return. It is subsequently considered to enhance stroke volume and arterial pressure [32], resulting in lower HR. As described above, a series of circulatory responses due to cold exposure eventually reduces sympathetic nerve activity while shifting autonomic heart rate control toward a parasympathetic dominance mediated by arterial baroreflex activation [33,35]. $\dot{V}$E, reflective of parasympathetic activity, was significantly lower in the WBC trial than the CON trial. The relative dominance of sympathetic or parasympathetic activity may affect gastric motility [36]. Gastric motility plays an important role in appetite regulation [36,37]. Wang et al. [38] indicated that stimulation of vagal cholinergic fibers and administration of acetylcholine stimulate gastric motility, suggesting close association between parasympathetic activity and gastric motility. Collectively, it is possible that the activation of the parasympathetic nervous system following WBC stimulates gastric motility, leading to increased energy intake in the present study.

### 4.3. Limitations

Since we specifically focused on the impact of post-exercise WBC treatment on energy intake during the subsequent meal, a baseline trial without exercise was not conducted. Therefore, it is inconclusive as to whether exercise-induced reduction of energy intake occurred in the present study. However, lowered energy intake following repeated sprint exercise is evident in some previous studies [3,9,10]. Moreover, athletes are susceptible to exercise-induced appetite suppression [39]. Thus, we speculate that energy intake in both the WBC and CON trials would be lower compared to a baseline condition without exercising. Furthermore, we did not measure other anorexigenic hormones, such as PYY_3-36_ and GLP-1. Although the influence of cold environment on these hormones remains unclear, WBC-induced elevation of energy intake may be partly mediated by alteration in these hormones. Further investigations are needed to explore the effect of post-exercise cold environment on these anorexigenic hormones. Interestingly, the greater rating of fullness was observed after meal in the WBC trial, suggesting that sensation for fullness by the meal might be changed after WBC. The determination of impact of post-exercise WBC on subjective perception for meal is also required.

### 4.4. Perspectives

From a practical viewpoint, the use of WBC is recommended as a novel post-exercise treatment because it has been shown to attenuate exercise-induced muscle damage and promote recovery of muscle function [23,40]. Therefore, increased energy intake following WBC treatment may assist physical recovery in addition to the anti-inflammatory effect of the WBC. The present findings provide valuable information, since insufficient energy intake during post-exercise periods may delay recovery of muscle energy substances (e.g., muscle glycogen, intramyocellular lipid content) and subsequently attenuate exercise performance.

## 5. Conclusions

In conclusion, 3 min of WBC treatment after high-intensity exercise increased subsequent energy intake during an ad libitum buffet meal test in well-trained athletes. It was unlikely that changes in appetite-regulating hormones mediated the augmented energy intake following post-exercise WBC treatment.

## Figures and Tables

**Table 1:** Score of subjective feeling of fatigue, muscle soreness and appetite.

		Pre	Post-Exercise			After Meal	Interaction (Trial × Time)	Main Effect	
			0 Min	15 Min	30 Min			Trial	Time
Fatigue (mm)	WBC	25 ± 20	87 ± 10 *	49 ± 25 *	48 ± 16 *	43 ± 15 *	0.288	0.058	<0.001
	CON	29 ± 17	87 ± 11 *	65 ± 19 *	59 ± 19 *	49 ± 18 *			
Muscle soreness (mm)	WBC	28 ± 22	56 ± 25 *	34 ± 17	37 ± 13	37 ± 15	0.210	0.032	<0.001
	CON	33 ± 22	66 ± 20 *	54 ± 18 *^,^†	48 ± 19 †	42 ± 20			
Hunger (mm)	WBC	66 ± 14	24 ± 15 *	47 ± 16 *	59 ± 20	9 ± 9 *	0.422	0.912	<0.001
	CON	66 ± 17	30 ± 18 *	42 ± 18 *	55 ± 16	13 ± 10 *			
Fullness (mm)	WBC	17 ± 16	44 ± 29 *	33 ± 19	24 ± 15	91 ± 8 *	0.005	0.881	<0.001
	CON	16 ± 13	50 ± 25 *	40 ± 22 *	30 ± 18	76 ± 23 *^,^†			
Prospective food consumption (mm)	WBC	63 ± 16	26 ± 21 *	44 ± 18 *	54 ± 19	12 ± 11 *	0.631	0.740	<0.001
	CON	62 ± 17	30 ± 17 *	42 ± 19 *	53 ± 18	19 ± 15 *			

Values are means ± standard deviation (*n* = 12). *: *p* < 0.05 vs. pre †: *p* < 0.05 vs. WBC. WBC: whole body cryotherapy; CON: rest period.

**Table 2:** Respiratory variables during post-exercise period in the WBC and CON trials.

	WBC	CON	*p*
$\dot{V}$O_2_ (mL/min)	349 ± 42	362 ± 48	0.196
$\dot{V}$CO_2_ (mL/min)	201 ± 55	240 ± 55 †	0.007
$\dot{V}$E (L/min)	11.3 ± 3.4	14.0 ± 4.2 †	0.016
HR beats per minute (bpm)	95 ± 11	101 ± 8 †	0.042

Values are means ± SD (*n* = 12). †: *p* < 0.05 vs. WBC. $\dot{V}$O_2_: oxygen uptake; $\dot{V}$CO_2_: carbon dioxide output; $\dot{V}$E: minute ventilation; HR: heart rate. WBC: whole body cryotherapy; CON: rest period.

**Table 3:** Blood glucose, lactate and serum insulin concentrations.

		Pre	Post-Exercise		Interaction (Trial × Time)	Main Effect	
			0 Min	30 Min		Trial	Time
Glucose (mmol/L)	WBC	5.0 ± 0.3	5.6 ± 2.4 *	5.1 ± 1.4	0.498	0.252	0.004
	CON	4.8 ± 0.4	6.0 ± 1.3 *	4.8 ± 1.1			
Lactate (mmol/L)	WBC	1.4 ± 0.3	20.5 ± 2.8 *	10.9 ± 2.5 *	0.871	0.995	<0.001
	CON	1.4 ± 0.4	20.3 ± 2.4 *	11.0 ± 2.4 *			
Insulin (pmol/L)	WBC	44 ± 8	125 ± 40 *	72 ± 66	0.518	0.748	<0.001
	CON	40 ± 14	129 ± 36 *	64 ± 40			

Values are means ± SD (*n* = 12). *: *p* < 0.05 vs. pre. WBC: whole body cryotherapy; CON: rest period.
